# Milk-derived bioactive peptides inhibit human endothelial-monocyte interactions *via* PPAR-γ dependent regulation of NF-κB

**DOI:** 10.1186/s12950-014-0044-1

**Published:** 2015-01-20

**Authors:** Simone Marcone, Karen Haughton, Paul J Simpson, Orina Belton, Desmond J Fitzgerald

**Affiliations:** FHI, Food for Health Ireland, University College Dublin, Dublin, Ireland; School of Medicine and Medical Science, UCD Conway Institute, University College Dublin, Belfield, Dublin, 4 Ireland; School of Biomolecular and Biomedical Science, UCD Conway Institute, University College Dublin, Dublin, Ireland; Teagasc, Biotechnology Centre, Moorepark, Fermoy, County Cork Ireland

**Keywords:** Inflammation, Atherosclerosis, Milk-derived bioactive peptides, NF-κB, PPAR-γ

## Abstract

**Background:**

Milk-derived bioactive peptides retain many biological properties and have therapeutic effects in cardiovascular disorders such as atherosclerosis. Under inflammatory conditions the expression of endothelial cells adhesion molecules is induced, increasing monocyte adhesion to human vessel wall, a critical step in the pathogenesis of atherosclerosis. In the present work we explored the effects of milk-derived bioactive peptides on the expression of the inflammatory phenotype of human endothelial cells and their effects on monocyte adherence to endothelial cells.

**Results:**

Treatment of endothelial cells with milk-derived hydrolysate inhibited their production of inflammatory proteins MCP-1 and IL-8 and expression of VCAM-1, ICAM-1 and E-selectin. Milk derived hydrolysate also attenuated the adhesion of human monocytes to activated endothelial cells. The effect was similar to that obtained in endothelial cells treated with troglitazone, a ligand of peroxisome proliferators-activator receptor-gamma (PPAR-γ). PPAR-γ is a transcription factor which when activated antagonises the pro-inflammatory capability of nuclear factor κB (NF-κB). We further examined whether the effects of milk-derived hydrolysates on endothelial cells may be mediated through NF-κB activation *via* a PPAR-γ dependent mechanism. The specific PPAR-γ inhibitor, GW9662 blocked the effects of the hydrolysate on the NF-κB-mediated chemokines and adhesion molecules expression in endothelial cells.

**Conclusions:**

These results suggest that milk-derived bioactive peptides work as anti-atherogenic agents through the inhibition of endothelial-dependent adhesive interactions with monocytes by inhibiting the NF-κB pathway through a PPAR-γ dependent mechanism.

**Electronic supplementary material:**

The online version of this article (doi:10.1186/s12950-014-0044-1) contains supplementary material, which is available to authorized users.

## Background

Atherosclerosis is a chronic inflammatory disease of arteries characterized by the formation of an atherosclerotic plaque, which is formed by abnormal deposition of lipids, infiltration of inflammatory cells and cellular proliferation [1-3]. There is growing evidence that consumption of low-fat milk and dairy product may be beneficial in the prevention or treatment of cardiovascular disease [4]. Elwood PC *et al.,* in a meta-analysis study showed that the consumption of dairy products is a protective factor for preventing ischemic vascular disease, stroke and diabetes [5]. Bovine milk contains a range of bioactive molecules such as lysozyme, lactoferrin, immunoglobulins, growth factors and hormones [6]. The beneficial effects of milk components and dairy products may be due to the biological properties of native proteins or to bioactive peptides derived from these proteins, making them potential ingredients of health-promoting foods [7].

Milk-derived bioactive peptides can be encrypted in both casein (α-, β- and γ-casein) and whey proteins (β-lactoglobulin, α-lactalbumin, serum albumin, immunoglobulins, lactoferrin, protease-peptone fractions). Casein hydrolysate containing peptides from casein is obtained in several ways, such as enzymatic hydrolysis or microbial fermentation, where proteolysis is by enzymes derived from microorganisms or plants [8,9]. These bioactive peptides may exert a range of physiological effects on the cardiovascular, digestive, endocrine, immune and nervous systems [10-12]. Several published studies have demonstrated the effects of milk constituents *in vitro* [13,14], while studies in animal and human models suggest that bioactive peptides derived from milk may have beneficial effects in cardiovascular disorders, reduce arterial stiffness and improve endothelial activity [15-17]. Endothelial dysfunction plays a central role in the initiation and pathogenesis of atherosclerosis [18,19]. Monocytes adhering to activated vascular endothelial cells (EC) and migrating into the extravascular space characterise the early inflammatory phase of atherogenesis [20]. Many stimuli (i.e. oxidised low-density lipoprotein cholesterol, diabetes mellitus, hypertension) injure and modify the vascular endothelium, increasing expression of adhesion molecules, such as VCAM-1, ICAM-1 and E-selectin, thus promoting vascular permeability and facilitating monocyte recruitment [21].

There are a number of endogenous pathways, including activation of the peroxisome proliferators-activator receptor-gamma (PPAR-γ), which regulate initiation and pathogenesis of atherosclerosis. Natural and synthetic agonists of PPAR-γ prevent endothelial cell activation and inflammatory cell adhesion in response to injury [22]. PPAR-γ acts as a transcription factor to suppress the signal transduction and consequent activation of pro-inflammatory transcription factors such as nuclear factor κB (NF-κB) [23]. PPAR-γ is just one of a nuclear hormone receptor superfamily, whose activities are regulated through the high affinity binding of a broad range of natural and synthetic ligands, including polyunsaturated fatty acids and prostaglandin derivatives [24,25]. PPAR-γ is expressed at high levels in adipose tissues and has been found in many other cells, including those in the vasculature such as endothelial cells [26]. PPAR-γ agonists may have anti-inflammatory and anti-atherogenic effects through the ability to inhibit several steps in the development of inflammation, in particular leukocyte infiltration into tissues mediated by NF-kB dependent expression of adhesion molecules [27-30].

This study explores the effects of casein-derived bioactive peptides on the expression of the inflammatory phenotype of EC and their effects on monocyte adherence to EC induced by TNF-α. We demonstrate that casein hydrolysate inhibits the pro-inflammatory NF-κB pathway through activation of PPAR-γ, reducing the expression of adhesion molecules and chemokine production in EC.

## Methods

### Materials

Recombinant human TNF-α was purchased from R & D Systems (McKinley Place, MN). GW9662, troglitazone and MTT (3-(4, 5-dimethylthiazolyl-2)-2,5-diphenyltetrazolium bromide) were purchased from Sigma-Aldrich (Dorset, UK). Anti-phospho-p65 (serine-536) antibody was purchased from Cell Signaling (Beverly, MA). All other reagents used in this study were of the highest purity and, unless otherwise stated, were obtained from Sigma-Aldrich (Dorset, UK).

### Preparation of the hydrolysate

*Enterococcus* strain DPC763 was originally isolated from an Irish dairy processing plant and deposited in the Moorepark Dairy Products Culture Collection (Teagasc, Cork, Ireland). The strain was routinely cultured in GM17 medium prepared from M17 (Oxoid, Basingstoke, UK) supplemented with 1% (w/v) glucose (Sigma-Aldrich, Dorset, UK). Prior to inoculation into 2.5% (w/v) sodium caseinate obtained from bovine milk (Kerry Food Ingredients) supplemented with 0.5% (w/v) glucose (Sigma-Aldrich, Dorset, UK), a single colony was cultured in 10 ml of GM17 and incubated aerobically for 24 h at 30°C under shaking conditions (100 rpm). A 1% (v/v) cell suspension was removed and added to fresh GM17 and incubated as above. Cells were then harvested by centrifugation (5000 rpm™ 15 min) and washed in an equal volume of sterile water, re-spun and suspended in water at a tenth of the original volume. The concentrated cell paste was added at 1% (v/v) to the sodium caseinate medium in a pH controlled bioreactor (set-point pH 6.5) set at 30°C with 100-rpm agitation for 40 h. 1 M NaOH was used for base addition. At the end of the incubation the material was heated to 80°C for 90 min in a water bath, cooled overnight at 4°C, transferred to a round-bottom flask, frozen during rotation in a methanol bath and stored at −20°C. Powders were produced using a Labconco freeze-dryer (Kansas City, USA) and the sample tested in the current study represents the product of four incubation reactions; powders were stored at 4°C. For experiments, the freeze-dried powders were reconstituted in ultra-pure water (20 mg/ml), centrifuged at 5000 rpm for 5 min and filter sterilized using 0.22 μm membrane. Samples prepared as described above but without added *Enterococcus* strain DPC763 were used as the control. Regenerated sample of the hydrolysate was obtained by incubating a different batch of sodium caseinate with a different batch of Enterococcus strain DPC763; the incubation of EC with the regenerated sample showed similar results to the data obtained with the original hydrolysate prepared previously, confirming the reproducibility of the procedure and of the study (Additional file 1: Figure S1).

### Gel permeation HPLC

Gel permeation HPLC of the *Enterococcus* incubated sodium caseinate medium were performed using a TSK G2000 SW column (600 × 7.5 mm, from Tosu Hass, Japan) fitted to a Waters (Millipore, Midlesex, UK) HPLC system. The column was run at a flow rate of 1 ml/min using 30% (v/v) acetonitrile containing 0.1% TFA as mobile phase and the eluant was continuously monitored at A_214_ using a Waters Model 441 fixed wavelength detector. Powder based samples were dissolved in sterile water at 25 mg/ml and left at 4°C overnight. All samples were filtered through a 0.22 μm filter prior to injection. Chromatographic data were collected and analyzed using a Minichrom™ data handling system (VG, Data Systems, Cheshire, UK). A molecular weight calibration curve was prepared from average retention volumes of standard proteins and peptides.

### Cell culture

Human aortic endothelial cells (EC) were obtained from Cascade biologics – Invitrogen cell culture (Carlsbad, CA) and were grown in “endothelial cell culture media MV” plus growth supplements from Promo Cell (Heidelberg, DE) and supplemented with 100 units/ml penicillin and 100 ug/ml streptomycin. For experiments, 80% confluent EC were incubated in endothelial cell basal media from Promo Cell (2% foetal calf serum, 0.4% endothelial cell growth supplements, 90 μg/ml heparin and with 100units/ml penicillin and 100 ug/ml streptomycin) for 24 h before treatments. Human THP-1 monocytes were purchased from the LGC Promochem (Middlesex, UK) and cultured in RPMI medium supplemented with 10% foetal bovine serum, L-glutamine (2 mM) and antibiotics (100 U/mL penicillin, 100 μg/mL streptomycin). Cells were grown in a humidified atmosphere of 5% CO_2_/95% air at 37°C. DMSO 3 μl in 2 ml of media was used as the vehicle control for each sample.

### Cell viability assay

EC were seeded in a 96-well tissue culture plate (2 × 10^4^ cells per well) for 24 h before the experiment. EC were then treated with various concentrations of sodium caseinate hydrolysates (3, 30, 300 and 600 μg/ml) for 18 h. After treatment, the medium was removed and 40 μl of fresh medium plus 10 μl of MTT solution (5 mg/ml in PBS, 0.22 μm filter sterilized) were added to the wells and incubated for 3 h at 37°C. Subsequently, 100 μl of DMSO was added into each well to dissolve the purple precipitate formed. The absorbance was recorded at 570 nm in a microtitre plate reader (Spectramax M2, Molecular devices, CA). The average values were determined from triplicate readings and the average value for the blank was subtracted from the samples. Cell viability assay was repeated in triplicate and the average value was expressed as a percentage of vehicle control.

### RNA isolation and Real-Time PCR analysis

Following treatments with sodium casein hydrolysate (18 h) and stimulation with TNF-α (0.5 ng/ml for 6 h) the EC were washed twice with PBS, before adding 300 μl of RLT buffer (Qiagen, UK). Total RNA was isolated from EC lysates using the RNeasy kit (Qiagen, UK). Reverse transcription was carried out on 1000 ng of total RNA using reverse transcriptase (Promega) according to the manufacturers’ instructions. Quantification of gene expression by real-time PCR was performed on an ABI Prism 7700 Sequence Detection System, (Applied Biosystems Inc., UK). VCAM-1, ICAM-1, E-sel, MCP-1 and IL-8 gene expression was examined using specific Taqman gene expression assays (Applied Biosystems Inc., UK). 18S was used as endogenous control in the assay. The mRNA levels were expressed as median values of three independent experiments. Casein hydrolysate incubation time (18 h) has been selected on the basis of a time-course experiment which showed that the maximal effect of inhibition of adhesion molecules expression was reached after 18 h of hydrolysate incubation (data not shown).

### Flow cytometric analysis of surface marker expression

Following treatments with casein-derived hydrolysate (18 h) and activation with TNF-α (0.5 ng/ml for 6 h), the EC were harvested to be stained for flow cytometric analysis. To block non-specific binding, EC were incubated in 2% BSA in PBS for 15 min before adding the antibodies. FITC-VCAM-1, PE-ICAM-1 (BD, Rankling Lakes, NJ) and APC-E-sel (Bio Legend, San Diega, CA) antibodies were used to label EC in 2% BSA in PBS. The antibodies were incubated for 30 min at RT. Following washes, the cells were fixed in 2% paraformaldehyde. Forward and side scatter gates were established to exclude nonviable cells and cell debris from the analysis. The mean fluorescence intensity of 2 × 10^4^ cells was analysed in each sample. Auto-fluorescence signals generated by unlabelled cells were used as negative controls in each experiment. Flow cytometric analysis was performed on an Accuri C6 instrument and analyzed with CFlow® Software (Accuri, Ann Arbor, MI). The protein expression levels were expressed as median fluorescence intensity of three independent experiments.

### Adhesion assay

For static adhesion assay, 4 × 10^4^ EC were seeded in a 96 well plate for 24 h and then treated with hydrolysates for 18 h. Subsequently, EC were stimulated with TNF-α (0.5 ng/ml) for 6 h. After treatments, the wells were washed three times with medium and 1 × 10^5^ fluorescein-labelled THP-1 monocytes were added to each well and incubated for 30 min at 37°C. After incubation, the wells were washed three times with medium and adherent monocytes were measured in a Spectramax M2 (Molecular devices, CA) plate fluorescence reader with 485 nm excitation and 530 nm emission wavelength. For photomicrographs, fluorescence-labelled adherent monocytes in the 96-well plate were analyzed using a Zeiss AxioImager M1 fluorescent microscope. Adhesion assay experiments were repeated in triplicate and the average value was expressed as a percentage of control (vehicle).

### Enzyme-linked immunosorbent assay

After treatment of EC with hydrolysates (18 h), the cells were washed and fresh medium was added to the wells; subsequently the cells were treated with 0.5 ng/ml TNF-α for 6 h and the supernatants subsequently collected and cleared by centrifugation (10,000 × g for 10 min at 4°C). The concentration of MCP-1 and IL-8 in conditioned media was determined by EIA using commercially available kits (MSD, Gaithersburg, MD) according to the manufacturer’s guidelines.

### Western blot analysis

Proteins extracted with lysis buffer (1% Nonidet P-40, 0.1% SDS, 150 mM NaCl, 50 mM Tris–HCl, pH 7.2) from treated EC were separated by sodium dodecyl sulfate polyacrylamide gel electrophoresis (SDS-PAGE) and blotted onto 0.45 uM nitrocellulose transfer membrane (Pall Life Sciences, New York, NY, USA). Membranes were blocked using 5% non-fat dried milk in TBS with 0.05% Tween (TBS-T) for 1 h and incubated overnight at 4°C with phospho-p65 and with βactin antibodies (Cell Signaling, Beverly, MA). Membranes were then washed with TBS-T and incubated for 1.5 h at room temperature with either peroxidase conjugated anti-mouse or anti-rabbit IgG (Cell Signaling, Beverly, MA). After further washing the proteins were visualized with super signal chemiluminescent reagent (Pierce, Northumberland, UK). Membranes were exposed to film and processed using an Agfa X-ray processor. Where required, membranes were stripped by incubating them in stripping buffer (Pierce, Northumberland, UK) for 30 min at RT, before probing with a subsequent antibody. Optical Density (OD) quantification of different specific bands was calculated using laser densitometry (Bio-Rad, Hertfordshire, UK) and normalized to the OD of β-actin.

### Statistical analysis

Results are expressed as mean ± SEM. Experimental points were performed in triplicate with a minimum of three independent experiments (n = 3). Statistical analysis of the dose–response experiments and statistical comparisons of 3 or more samples were carried out using one-way ANOVA. When a significant overall effect was detected, a Dunnett post hoc correction was used for multiple comparisons. Statistical comparison of one condition versus control was made by using Student’s unpaired *t*-test assuming unequal variance. A statistical value of *P < 0.05 or greater was considered significant. There was no significant difference between cells alone (untreated) and DMSO (vehicle control) treated cells; therefore, DMSO was used as the reference treatment.

## Results

### Peptide size distribution of the sodium casein-derived peptides and effects on endothelial cell viability

**Figure 1:**
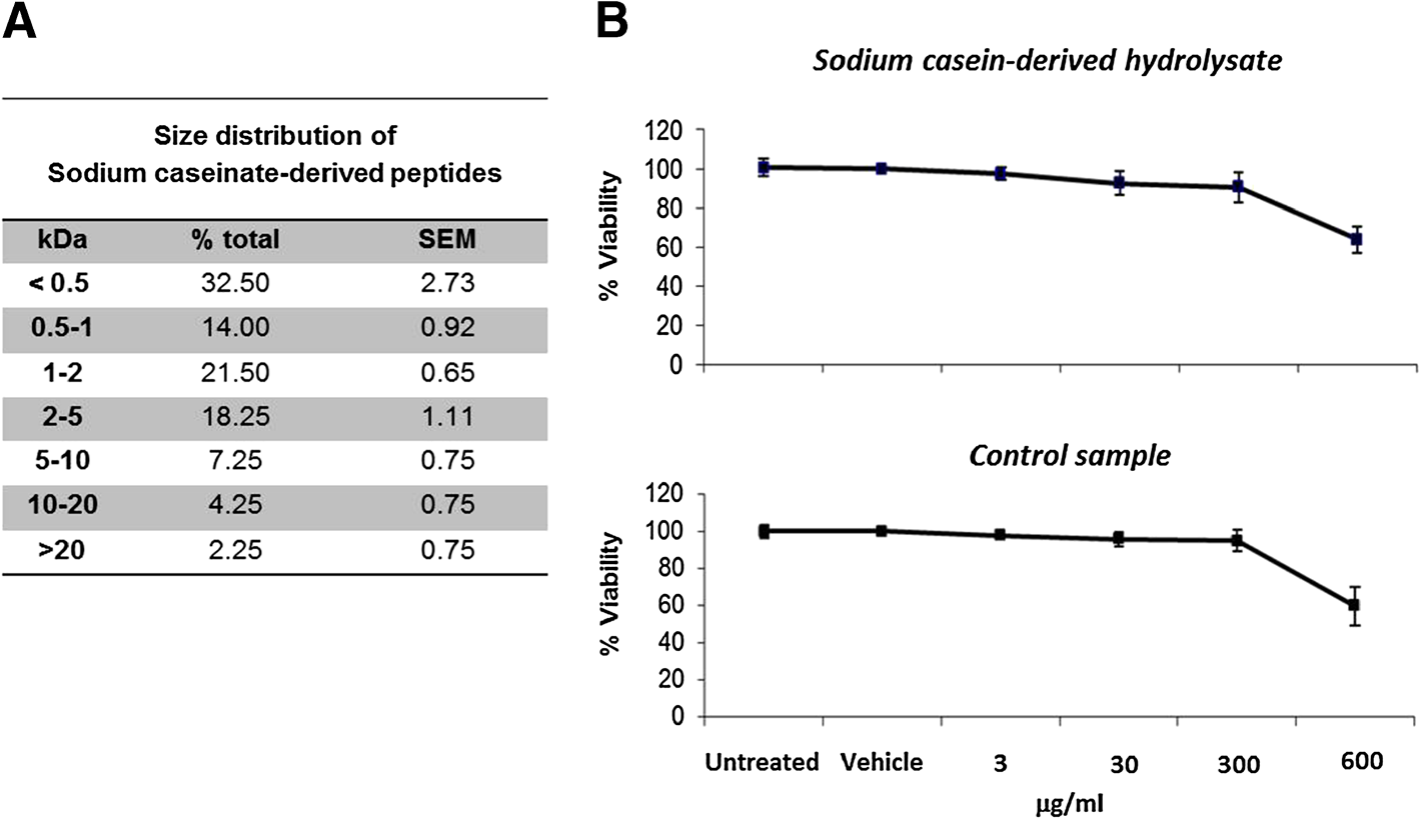
**Peptide size distribution of the sodium casein-derived peptides and effects on endothelial cell viability** ***.*** **(A)** Gel permeation chromatography of the sodium casein-derived peptides was performed to assess the peptide size distribution of the hydrolysate. Data show the mean ± SEM of four incubation reactions. **(B)** MTT assay was carried out to determine the cell viability of EC treated with casein hydrolysate or with the control sample for 18 h, resulting in more than 94% ±3.4% viable cells up to a concentration of 300 μg/ml. Data are expressed as mean ± SEM of 3 independent experiments. Data were reported as percentage of control (untreated cells).

A gel Permeation HPLC analysis of the *Enterococcus* incubated sodium caseinate medium was performed to assess the peptide size distribution of the hydrolysate, obtained from four incubation reactions pooled to generate the respective hydrolysate powder. Most of the peptides (86.2%) resulted with a mass <5 kDa, while only 2.2% with a mass >20 kDa (Figure 1A). A gel Permeation chromatography profile of the control sample showed that the mass of its components was >20 kDa, confirming the generation of sodium caseinate-derived peptides in the *Enterococcus* incubated sodium caseinate fermentate.

We then studied the effects of the samples on the EC viability. The incubation of EC with sodium casein hydrolysate for 18 h resulted in more than 94% ±3.4% viable cells at concentrations of 300 mg/ml (Figure 1B), which indicates that concentrations from 3 to 300 μg/ml do not significantly decrease cell viability, while at a concentration of 600 μg/ml the number of viable cells fell to 60% ±5.8%. A similar response was obtained incubating EC with the control sample, resulting in a cell viability of 91% ±4.5% up to 300 μg/ml. Thus, for these experiments we performed dose response assays using hydrolysate concentration up to 300 μg/ml.

### TNF-α-induced adhesion molecules expression is reduced by the sodium casein-derived peptides in EC

**Figure 2:**
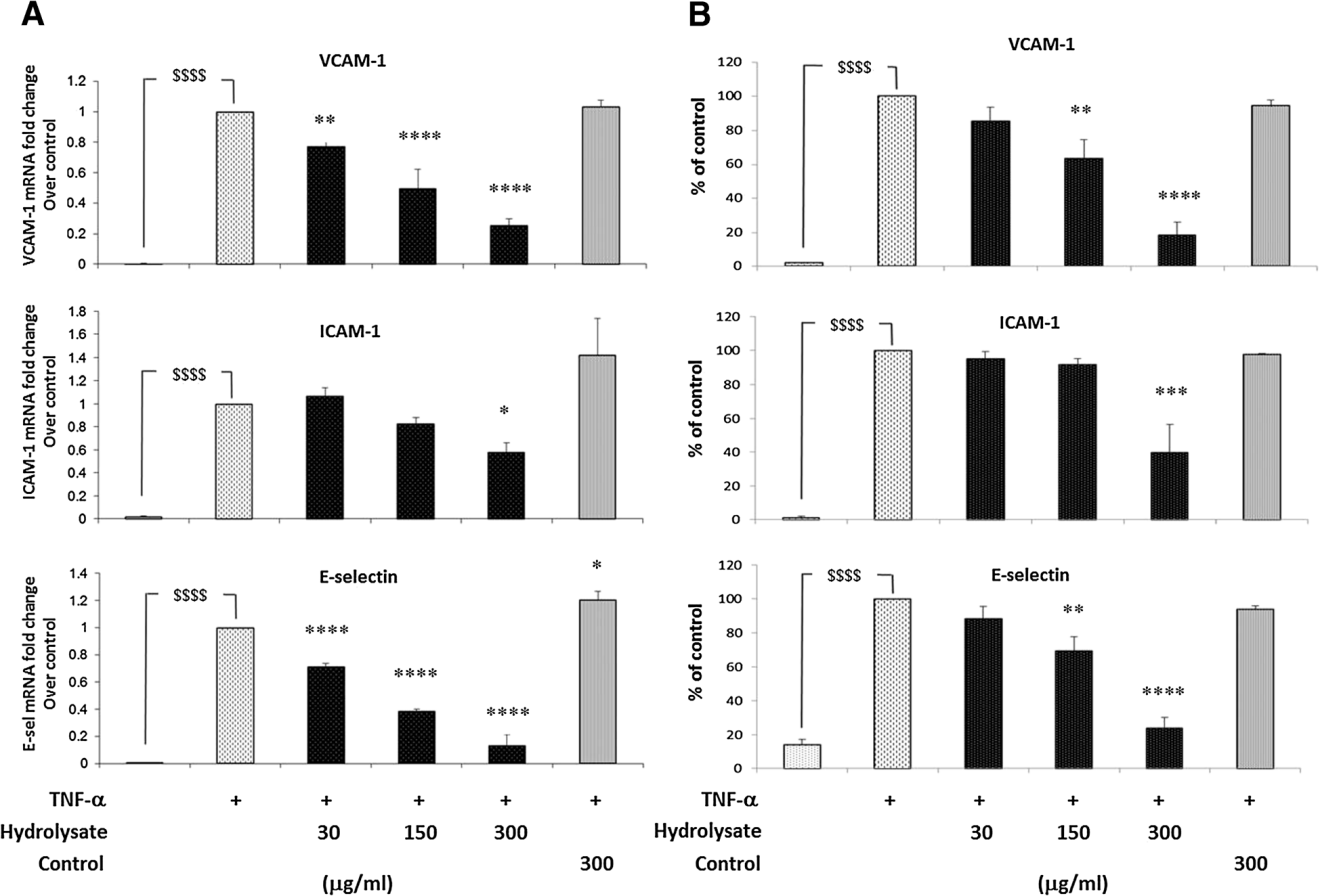
**TNF-α-induced adhesion molecules expression is reduced by sodium casein-derived peptides in EC. (A)** VCAM-1, ICAM-1 and E-selectin gene expression was performed on EC treated with casein hydrolysate or control sample for 18 h, followed by 6 h stimulation with TNF-α (0.5 ng/ml). Cells were harvested in RLT buffer, RNA was extracted and reverse transcription was performed for RT-PCR analysis. **(B)** For the quantification of protein surface expression of adhesion molecules, flow cytometric analysis was performed on TNF-α activated EC (6 h, 0.5 ng/ml) pre-incubated with casein hydrosylate. Data are expressed as mean ± SEM of 3 independent experiments. Statistical analysis was carried out using one-way ANOVA employing Dunnett correction for multiple comparisons. A statistical value of *P < 0.05 or greater was considered significant; $$$$ (p < 0.0001) vehicle vs. control; ****P < 0.0001, ***P < 0.001 and **P < 0.01 treatments vs. control (TNF-α activated EC).

To examine the effect of the hydrolysate on adhesion molecule expression in EC stimulated by TNF-α, EC were pre-treated with the hydrolysate or with the control sample for 18 h before adding TNF-α (0.5 ng/ml) for 6 h. As shown in Figure 2A, TNF-α enhanced VCAM-1, ICAM-1 and E-sel mRNA while the pre-treatment with the hydrolysate significantly reduced the expression of VCAM-1 (by 23% ±3%, 51% ±7% and 75% ±2%), ICAM-1 (by 0% ±4%, 18% ±3% and 43% ±5%) and E-sel (by 30% ±2%, 62% ±1% and 87% ±4%) in a dose dependent manner (with 30, 150 and 300 μg/ml of hydrolysate, respectively). The control sample had no effect on the gene expression of the adhesion molecules (dose–response experiments using the control sample are reported in Additional file 2: Figure S2). To confirm the gene expression results, we analysed the cell surface expression of adhesion molecules in EC pre-treated with the hydrolysate or with the control sample for 18 h and subsequently with TNF-α (0.5 ng/ml) for 6 h. As shown in Figure 2B, flow cytometric analysis showed a significant dose dependent (30, 150, nand 300 μg/ml) decrease of EC surface protein levels of VCAM-1 (by 15% ±8%, 37% ±11% and 82% ±8%), ICAM-1 (by 5% ±4%, 9% ±3% and 61% ±13%) and E-sel (by 22% ±7%, 31% ±8% and 77% ±6%). The control sample had no significant effect on the protein expression of the adhesion molecules. Taken together, these results suggest that the ability of the sample to modulate the gene and the cell surface expression of adhesion molecules is due to the bioactive peptides contained in the hydrolysate generated by the bacterial hydrolysis of sodium caseinate (Figure 2A).

The fermentation process used to produce the hydrolysate is a pH controlled reaction and sodium hydroxide is added to compensate for the organic acid produced by the *Enterococcus* strain. This will lead to the formation of sodium lactate in the final powder (~25 mg/g) that is not in the control sample. Thus, we used two different samples obtained from the fermentation of sodium caseinate with other *Enterococcus* strains, to compare to the hydrolysate studied. As shown in Additional file 2: Figure S2, these samples do not have any significant effect on adhesion molecules expression in EC, demonstrating that the effects of the hydrolysate studied here is due to the specific bioactive peptides generated by the bacterial fermentation with *Enterococcus* strain DPC763 and the effects are not due to a generally hydrolysed material or pH effect.

### Adhesion of human monocytes to TNF-α activated EC is prevented by sodium casein-derived peptides

**Figure 3:**
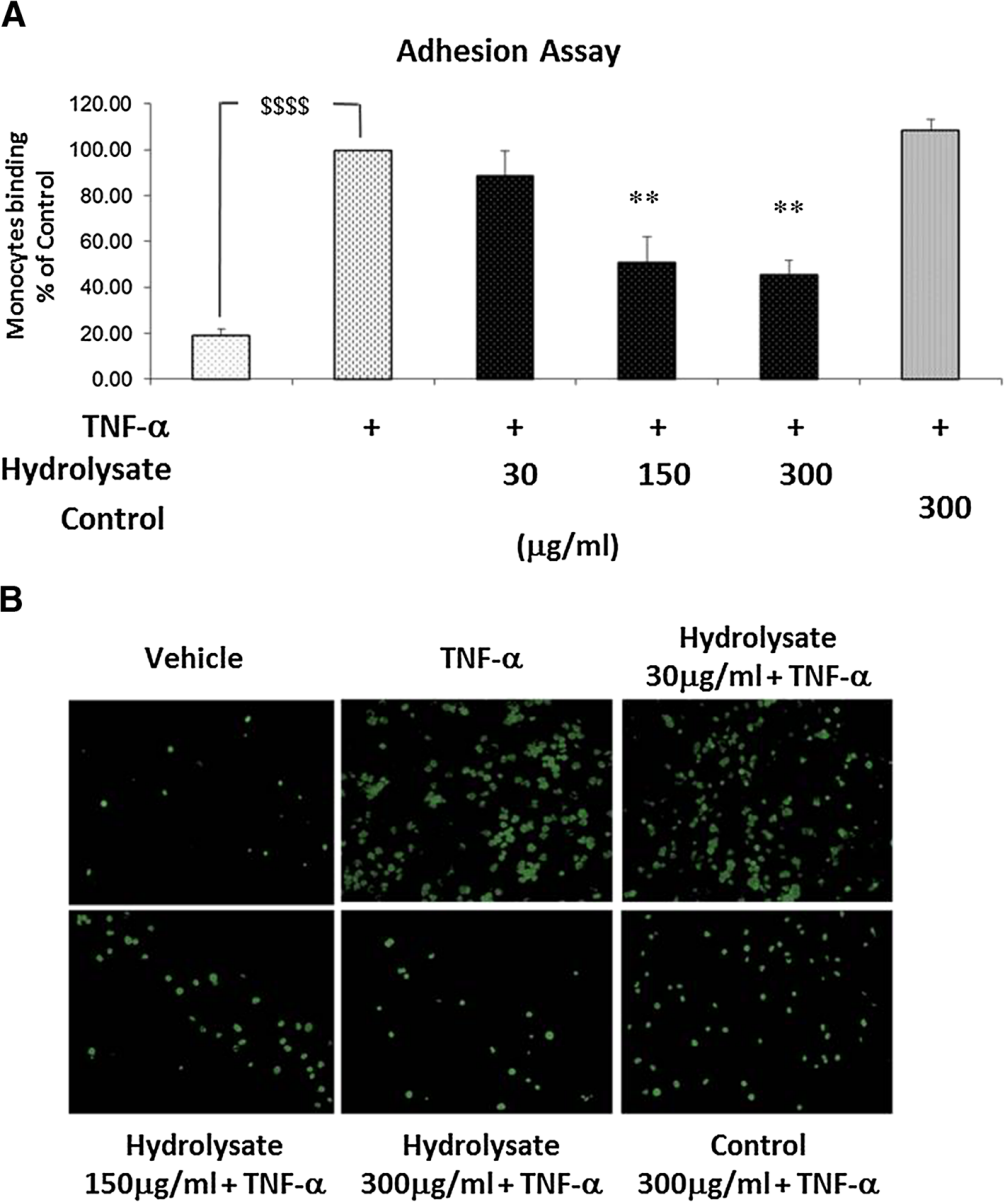
**Adhesion of human monocytes to TNF-α activated EC is prevented by sodium casein-derived peptides.** EC were treated with samples for 18 h, followed by 6 h stimulation with TNF-α 0.5 ng/ml) and a static adhesion assay with fluorescence-labelled THP-1 human monocytes was performed. **(A)** Adherent monocytes were measured in a plate fluorescence reader with 485 nm excitation and 530 nm emission wavelength. Data were calculated as mean +/− SEM of 3 independent experiments. Statistical analysis was carried out using one-way ANOVA employing Dunnett correction for multiple comparisons. A statistical value of *P < 0.05 or greater was considered significant; $$$$ (p < 0.0001) vehicle vs control; **P < 0.01 treatments vs control (TNF-α activated EC). **(B)** Representative fluorescence microscopy photomicrographs of monocytes adhesion to EC are shown: vehicle (top left); TNF-α (top centre); hydrolysate 30 μg/m l+ TNF-α (top right); hydrolysate 150 μg/ml + TNF-α (bottom left); hydrolysate 300 μg/ml + TNF-α (bottom centre); control 300 μg/ml + TNF-α (bottom right).

We examined the effects of the hydrolysate on the adhesion of the human monocyte cell line (THP-1 cells) to EC monolayers following treatment with the hydrolysate and TNF-α activation. As shown in Figure 3, TNF-α stimulation significantly increased the adhesion of THP-1 cells to EC (81% ±2%). Pre-treatment of the EC with hydrolysate inhibited the adhesion of THP-1 cells to TNF-α activated EC (by 22% ±10%, 49% ±11% and 55% ±6% with 30, 150 and 300 μg/ml of hydrolysate, respectively) in dose response manner. On the contrary, the adhesion of THP-1 cells was unchanged by the control sample, suggesting a critical role of the bioactive peptides generated in the hydrolasate in reducing monocyte cell-line adhesion.

### Sodium casein-derived peptides attenuate the TNF-α-induced pro-inflammatory profile in EC

**Figure 4:**
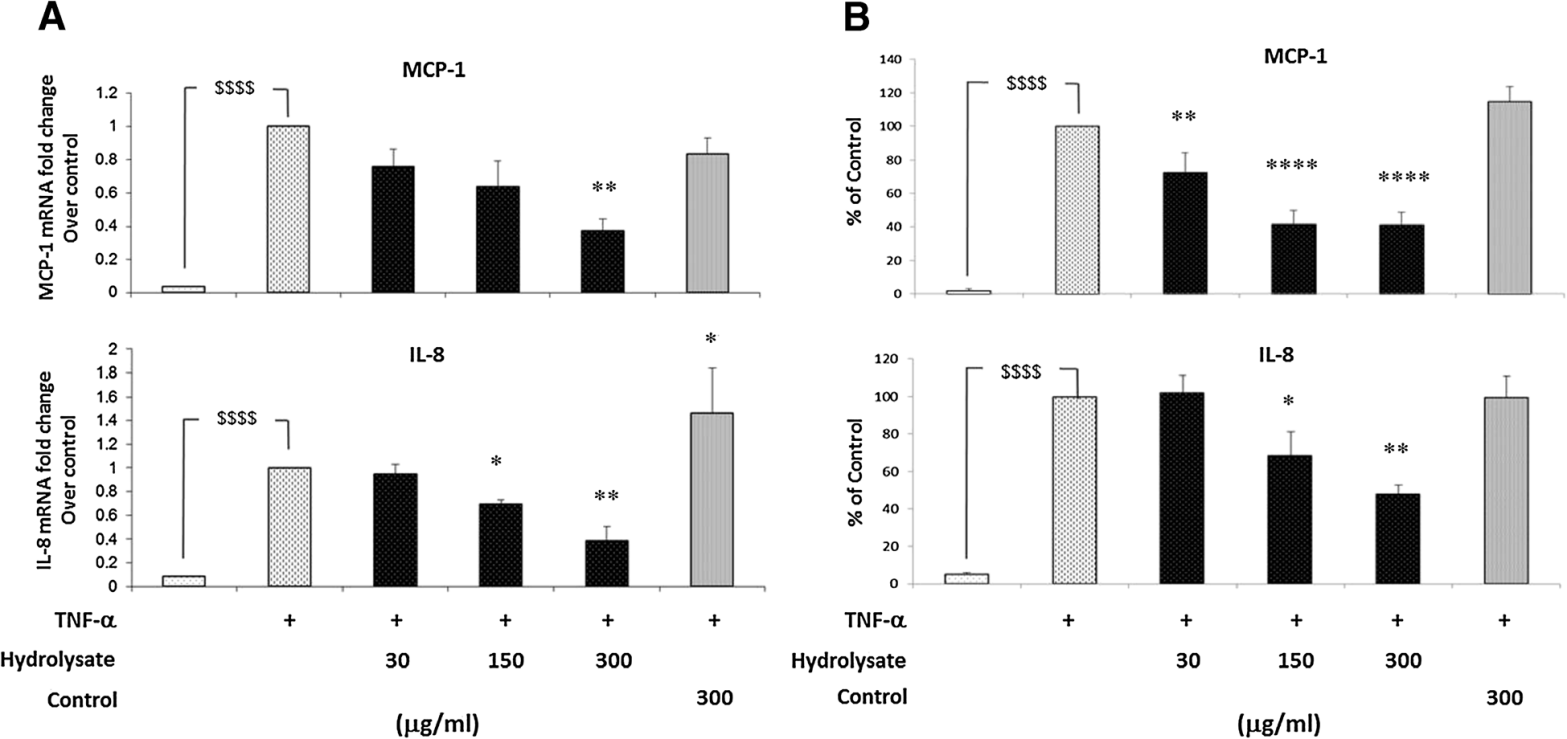
**Sodium casein-derived peptides attenuate the TNF-α-induced production of pro-inflammatory chemokines in EC. (A)** Gene expression levels of MCP-1 and IL-8 were measured in EC treated with casein hydrolysate or control sample for 18 h, followed by 6 h stimulation with TNF-α (0.5 ng/ml). Cells were harvested in RLT buffer, RNA was extracted and reverse transcription was performed for RT-PCR analysis. **(B)** The media from EC treated with the casein hydrolysate and activated with TNF-α was assayed to measure MCP-1 and IL-8 concentration by ELISA. Each experiment was carried out independently three times. Data were calculated as mean +/− SEM of 3 independent experiments and reported as percentage of control (TNF-α activated EC). Statistical analysis was carried out using one-way ANOVA employing Dunnett correction for multiple comparisons. A statistical value of *P < 0.05 or greater was considered significant; $$$$ (p < 0.0001) vehicle vs control; ****P < 0.0001, ***P < 0.001, **P < 0.01 and *P < 0.05 treatments vs control (TNF-α activated EC).

Given the critical role of MCP-1 and IL-8 as chemotactic inflammatory factors in the recruitment of monocytes following endothelial cells injury [31], we examined whether the hydrolysate could modulate the production of these chemokines in EC. In these experiments (Figure 4A), the hydrolysate significantly suppressed MCP-1 (by 25% ±10%, 37% ±15% and 63% ±6% with 30, 150 and 300 μg/ml of hydrolysate, respectively) and IL-8 (by 8% ±4%, 31% ±2% and 62% ±7% with 30, 150 and 300 μg/ml of hydrolysate, respectively) gene expression induced in EC treated with TNF-α. As shown in Figure 4B, the hydrolysate also decreased EC production of MCP-1 (by 28% ±6%, 59% ±4% and 59% ±4% with 30, 150 and 300 μg/ml of hydrolysate, respectively) and IL-8 (by 0% ±9%, 32% ±12% and 53% ±5% with 30, 150 and 300 μg/ml of hydrolysate, respectively) measured by ELISA. This reduction in MCP-1 and IL-8 production, together with the reduction in adhesion molecule expression explains the inhibition of monocyte adhesion to TNF-α activated EC.

### Sodium casein-derived peptides modulate the expression of pro-inflammatory phenotype of EC via PPAR-γ dependent mechanisms

**Figure 5:**
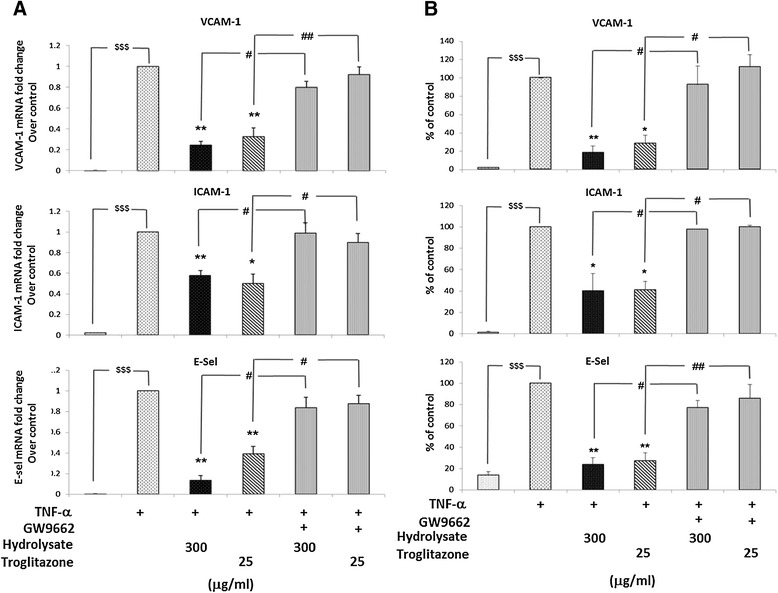
**Sodium casein-derived peptides modulate the expression of pro-inflammatory phenotype of EC via PPAR-γ dependent mechanisms.** TNF-α (0.5 ng/ml, 6 h) induced VCAM-1, ICAM-1 and E-sel expression was blocked by the PPAR-γ ligand, troglitazone (25 μM), similarly to the casein hydrolysate (300 300 μg/ml). In the presence of GW9662 (10 μM), a PPAR-γ antagonist, the effects of both troglitazone and the casein hydrolysate were completely reversed. **(A)** Gene expression levels of VCAM-1, ICAM-1 and E-sel were measured in EC treated with casein hydrolysate or troglitazone for 18 h and with or without GW9662 (10 μM), followed by 6 h stimulation with TNF-α (0.5 ng/ml). **(B)** Quantification of protein surface expression of adhesion molecules was carried out by flow cytometric analysis. Data are expressed as mean ± SEM of 3 independent experiments. Data were reported as percentage of control (TNF-α activated EC). Statistical comparison of one condition versus control was made by using Student’s unpaired *t*-test assuming unequal variance; $$$ (p < 0.001) vehicle vs. control; **P < 0.01 and *P < 0.05 treatment vs. control; ^##^P < 0.01 and ^#^P < 0.05 treatment vs. GW9662.

PPAR-γ is a nuclear receptor that suppresses inflammatory gene expression when activated and the inflammatory response in EC [32,33]. Thus, the PPAR-γ ligand, troglitazone, limits induction of adhesion molecules in EC as well as inflammatory cell adhesion to endothelial cells *in vitro* [22]. Consequently, we examined the role of PPAR-γ in the anti-inflammatory effects of the casein hydrolysate. Specifically, we examined whether the effects of casein hydrosylate in suppressing the inflammatory phenotype of activated EC were blocked by the specific PPAR-γ inhibitor, GW9662. EC were treated for 18 h with the hydrolysate or with the PPAR-γ agonist troglitazone (25 μM) in the presence or absence of GW9662 (10 μM) and then activated with TNF-α (0.5 ng/ml for 6 h). As shown in Figure 5, troglitazone significantly reduced the gene expression of VCAM-1 (by 68% ±8%), ICAM-1 (by 50% ±8%) and E-sel (by 62% ±7%) and the protein level of VCAM-1 (by 72% ±9%), ICAM-1 (by 60% ±8%) and E-sel (by 73% ±8%). Similarly, the casein hydrolysate reduced the gene expression of VCAM-1 (by 75% ±2%), ICAM-1 (by 43% ±5%) and E-sel (by 87% ±4%) and the protein level of VCAM-1 (by 82% ±8%), ICAM-1 (by 61% ±13%) and E-sel (by 77% ±6%) assessed by flow cytometry analysis. In the presence of GW9662, the effects of both troglitazone and the casein hydrolysate were prevented. These data suggest that the modulation of TNF-α-induced adhesion molecules response by casein derived bioactive peptides is mediated by activation of PPAR-γ.

### Casein hydrolysate exerts its anti-inflammatory effects by suppressing NF-κB pathway activation through PPAR-γ dependent mechanisms

**Figure 6:**
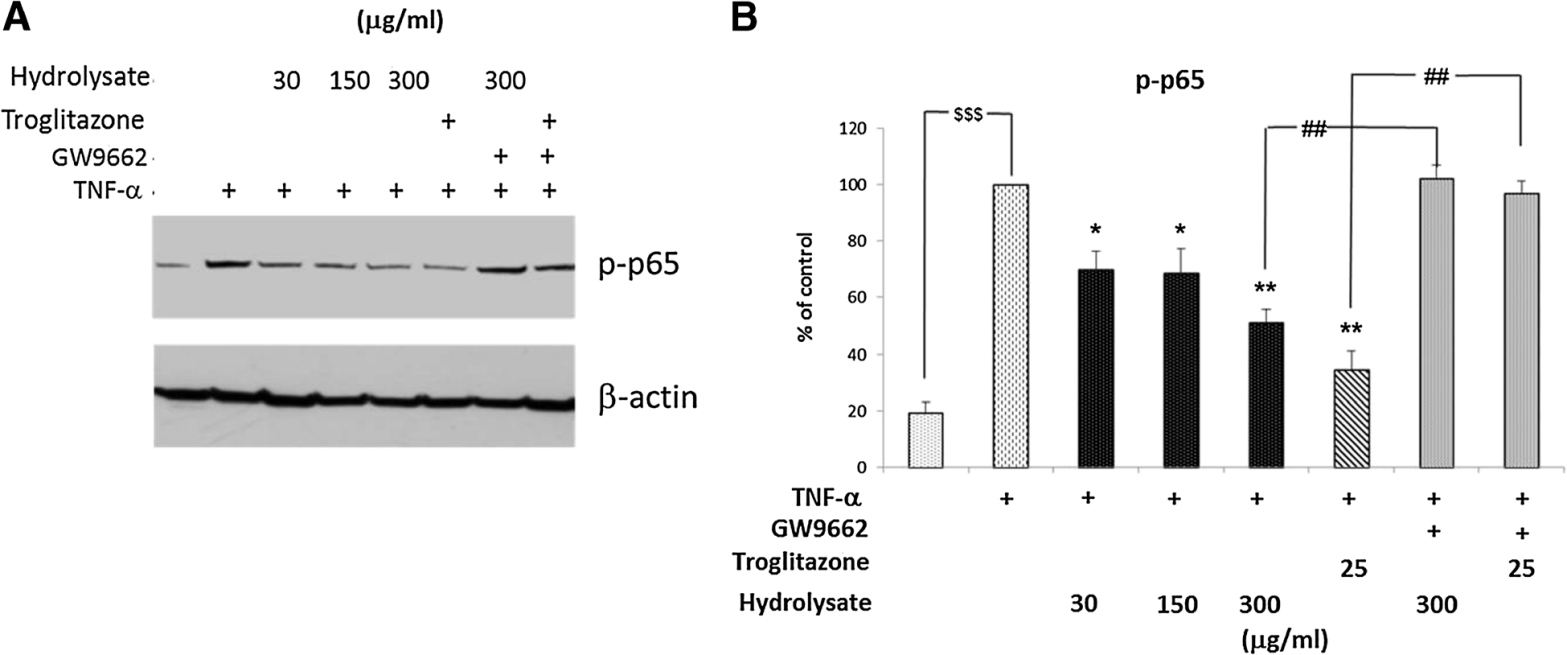
**Casein hydrolysate exerts its anti-inflammatory effects by suppressing NF-κB pathway activation through PPAR-γ dependent mechanisms.** Phosphorylation of the p65 subunit of NF-κB was used as an index of NF-kB activation. **(A)** EC were treated with casein hydrolysate or troglitazone (25 μM) for 18 h and with or without GW9662 (10 μM), followed by 10 min stimulation with TNF-α (0.5 ng/ml). Phosphorylation of p65 was estimated by Western blotting experiments (20 μg of total proteins) and β-actin was used as loading control. **(B)** Protein levels (assessed as ratio of protein OD normalized to the OD of β-actin) were shown as percentage of control (TNF-α activated cell) (n = 3). Statistical comparison of one condition versus control was made by using Student’s unpaired *t*-test assuming unequal variance; $$$ (p < 0.001) vehicle vs control; **P < 0.01 and *P < 0.05 treatment vs control; ^##^P < 0.01 treatment vs GW9662.

The expression of adhesion receptors is known to depend on the activation of NF-κB, following TNF-α stimulation [34]. To explore the hypothesis that the hydrolysate exerts its effects in EC *via* NF-κB through activation of PPAR-γ, EC were treated for 18 h with the hydrolysate or troglitazone (25 μM) in the presence or absence of GW9662 (10 μM) and then activated with TNF-α (0.5 ng/ml for 10 min), and EC lysates analysed by Western blotting. Phosphorylation of the p65 subunit of NF-κB was used as an index of NF-kB activation. As shown in Figure 6, TNF-α activates the NF-κB pathway inducing the phosphorylation of NF-κB p65 (p < 0.001, Figure 6A,B). Phosphorylation of NF-κB p65 was significantly reduced by hydrolysate 300 μg/ml (p < 0.01, Figure 6B) in a dose dependent manner and by the PPAR-γ ligand troglitazone (p < 0.01, Figure 6B). In the presence of GW9662, the effects of both the casein hydrolysate and troglitazone on NF-κB p65 phosphorylation (p < 0.01, Figure 6B) were completely suppressed, demonstrating that the modulation of NF-κB activation in EC by the casein hydrolysate was mediated by a PPAR-γ dependent mechanism.

## Discussion

Food-derived biological active peptides found in many different foods, including bovine milk, exert a physiological effect in humans. The bioactive peptides are inactive in their parent protein, but can be formed by enzymatic or bacterial hydrolysis during gastrointestinal digestion or during food processing (e.g., milk fermentation). These are usually short residues (2–20 amino acids), but may have more than 20 amino acids in size. Milk proteins, including casein, are currently the main source of several biofunctional peptides. The casein composition of bovine milk can vary due to several factors, such as, breed [35], stage of lactation [36], season [37], health [38] and genetic polymorphism [39]. At present, it is unknown if the bioactivity reported in our study may be affected by such changes, but the hydrolysate used in the current study was generated from sodium caseinate powder, and not from milk, and, on account of this, the possible variations in casein composition of bovine milk may hardly affect our hydrolysate composition.

Numerous products containing bioactive peptides are already on the market and casein derived peptides have already found interesting applications as dietary supplements and as pharmaceutical preparations [40]. Biofunctional peptides exhibit activities that may be beneficial in cardiovascular diseases [41,42] such as control of blood pressure, cholesterol lowering, suppression of free radical formation and effects on platelet [43].

Endothelial dysfunction is a critical element in the pathogenesis of atherosclerosis [18,19]. Endothelial cell activation induces the expression of inflammatory cytokines and adhesion molecules which stimulates leukocyte homing, adhesion and migration into the subendothelial space; these processes are fundamental to atherosclerotic lesion initiation and progression [3]. In the present study we reported a novel anti-inflammatory bioactivity of the casein hydrolysate derived by *Enterococcus* fermentation. We found that bioactive peptides derived from *Enterococcus* bacterial fermentation of sodium caseinate significantly inhibited TNF-α-induced expression of adhesion molecules as well as pro-inflammatory chemokine production in EC. To assess the mechanism of these effects of casein hydrolysate, we investigated the expression of mRNA for the genes of E-selectin, vascular cell adhesion molecule 1 and intercellular adhesion molecule 1, their surface expression after stimulation with TNF-α and the production of pro-atherogenic chemokines (MCP-1 and IL-8). The gene and protein expression of these pro-inflammatory mediators and the release of chemokines in TNF-α-activated EC were significantly inhibited by the treatment of EC with casein hydrolysate, and these effects were accompanied by inhibition of the adhesion of the human monocyte cell-line THP-1. VCAM-1, and ICAM-1 and E-sel are well-known inflammatory mediators involved in pathogenesis of atherosclerosis [44]. The levels of these molecules also increase in association with cardiovascular risk factors, and they have been correlated with measures of atherosclerotic plaque disease and with adverse cardiovascular prognosis [45,46]. Cell adhesion molecules may derive from multiple cellular types, but E-selectin is the most specific marker for endothelial cell activation [47], thus, our results suggest that the casein hydrolysate may inhibit cell adhesion molecules expression by inhibiting the activation of endhothelial cells.

Similar effects to the adhesion molecules inhibition by casein hydrolysate were found with troglitazone, a well-characterised PPAR-γ agonist. PPAR-γ is a negative regulator of inflammatory processes such as adhesion molecule expression and inflammatory chemokine production in EC [28-30]. Given that casein hydrolysate suppressed adhesion molecules and chemokine production, we further examined whether this was due to PPAR-γ activation. We investigated whether the effects of casein hydrolysate on EC were suppressed by the specific PPAR-γ antagonist, GW9662, and found that the inhibitory effects of casein hydrolysate on adhesion molecules expression were completely prevented when the EC were treated with GW9662. Similarly, GW9662 prevented the anti-inflammatory effects troglitazone in TNF-α treated EC. The inhibitory effect of PPAR-γ on EC inflammatory phenotype is mediated in part through NF-κB [48], a key transcription factor in EC activation and function [34]. In resting condition, NF-κB is maintained inactive in the cytosol of cells forming a complex with its inhibitor subunit [49]. Upon stimulation by cytokines, active NF-κB is free to enter the nucleus and to regulate the transcription of several pro-inflammatory genes, such as VCAM-1, ICAM-1, E-selectin, MCP-1 and IL-8 in EC. The phosphorylation of the p65 subunit of NF-κB on serine-536 is an important step in the activation of the NF-κB pathway *via* activation of several kinases including IκB kinases (IKKs) [50]. Therefore, the effects of casein hydrolysate and troglitazone on the phosphorylation of p65 subunit were examined as an index of NF-kB pathway activation. Our results showed that the PPAR-γ ligand troglitazone suppressed the activation of NF-κB by TNF-α in EC, decreasing the phosphorylation of p65 (Figure 6). The casein hydrolysate induced similar responses in a dose dependent manner. Moreover, GW9662 suppressed the effects of both troglitazone and the hydrolysate, suggesting a role for PPAR-γ in the NF-κB pathway modulation by the casein hydrosylate. Given that NF-κB plays a role in regulating the expression of adhesion molecules in EC, our data on the suppression of NF-κB suggests that casein hydrolysate may exert its inhibitory effects on adhesion molecules and chemokine expression through NF-κB. Further evidence that the effects of the casein hydrosylate are mediated by PPAR-γ is provided by experiments demonstrating that its effects on VCAM-1, ICAM-1 and E-sel expression are suppressed in the presence of the PPAR-γ antagonist GW9662.

It has been reported that other PPAR-γ activators, such as cyclopentenone prostaglandins, might also exert anti-inflammatory effects through suppression of NF-κB activation in a PPAR-γ-independent manner *via* direct inhibition of other steps of NF-κB pathway, such as IκB kinase [51]. It has been also reported that PPAR-γ ligands may have anti-inflammatory effects in a PPAR-γ and NF-κB independent manner interfering with other pathways such as activation of ERK [52] or inhibition of AP-1 pathways [53]. As the hydrolysate used in this study is a complex mixture of peptides of different chain length (Figure 1A), we cannot exclude the possibility that, together with the direct effect on NF-κB through PPAR-γ we showed here, the hydrosylate might exert its anti-inflammatory effect by acting at other levels of NF-κB activation or even by modulating other pathways. As shown in Additional file 3: Figure S3, gene expression analysis of adhesion molecules in EC treated with fractions of the sodium caseinate-derived hydrolysate showed that the fractions 0.5-5 KDa are the most bio-active, suggesting that the anti-inflammatory activity of the hydrolysate may be confined to the bio-active peptides present in that fraction. However, further study is required to specifically identify the functional components of the hydrolysate. Many of the known bioactive peptides are multifunctional and can exhibit different bioactivities, such as immunomodulation or antimicrobial, antioxidant, antithrombotic, cholesterol lowering, and antihypertensive properties [54-57]. Hypertension and thrombosis may be controllable risk factors in the development of cardiovascular diseases. Platelet activation and aggregation are central events in thrombus formation, and dysregulation of platelet physiology can contribute, together with endothelial dysfunction, to the pathogenesis of thrombotic events associated with hypertension [58,59]. Bioactive casein peptides have been reported to have functional effects on the cardiovascular system due to anti-thrombotic [60], anti-hypertensive [61] and anti-obesity [62] effects, suggesting a potential role in the control of cardiovascular diseases [43]. Yamamoto [9] and Maeno [63] described an anti-hypertensive effect of casein hydrolysates by using extracellular *Lactobacillus* proteases, and, in an another study, a sour milk product fermented with *L. Helveticus* and *Saccharomyces cerevisiae* reduced arterial blood pressure in rats [64] and humans [65]. Endothelial dysfunction is a central element in the pathogenesis of atherosclerosis, and some studies in animal and human models suggested that bioactive peptides derived from β-casein (Val-Pro-Pro and Ile-Pro-Pro), already known for their anti-hypertensive properties, can reduce arterial stiffness and improve endothelial functions [17,66,67]. Interestingly, a recent study showed potential anti-inflammatory property of tryptic hydrolysates of β-casein, mediated by the modulation of NF-κB [13]; together with our study, it describes an anti-inflammatory mechanism of action of casein hydrolysates. In addition, in the present study, we reported an anti-atherogenic bioactivity of the casein hydrolysate, and we demonstrated that the hydrolysate acts by inhibiting transcription factors (NF-κB and PPAR-*γ*) that are involved in the regulation of several inflammatory pathways which, in turn, are common to many diseases. Therefore, our results may offer a useful basis to understand the molecular mechanisms which may regulate many bioactivities attributed to milk derived bioactive peptides.

## Conclusions

Our findings demonstrate that the hydrolysate obtained by bacterial fermentation of sodium-caseinate suppresses the NF-κB pathway and in turn inhibits the expression of an EC inflammatory phenotype induced by TNF-α, by down-regulating inflammatory-cell adhesion molecule and chemokines expression. These effects were accompanied by inhibition of the adhesion of a human monocyte cell-line to TNF-α-activated human EC. This inhibitory effect on TNF-α-induced inflammation was completely reversed in the presence of the specific PPAR-γ inhibitor, GW9662, suggesting that the casein hydrolysate component(s) may be a ligand for PPAR-γ and through this mechanism inhibit NF-κB activation. Further study is required to explore the potential for milk protein hydrolysate in human atherosclerosis.

## Additional files

Additional file 1: Figure S1. Regenerated Sample of the Hydrolysate was obtained by incubating a different batch of sodium caseinate with a different batch of Enterococcus strain DPC763; the fermentation process was carried out as described in the Methods section. The incubation of EC with the regenerated sample showed similar results to the data obtained with the hydrolysate previously studied here, confirming the reproducibility of the procedure and of the study. (A) Gene expression analysis of adhesion molecules VCAM-1, ICAM-1 and E-Sel in EC treated with regenerated sample of the hydrolysate. EC were treated with samples for 18 h, followed by 6 h stimulation with TNF-α (0.5 ng/ml). (B) Adhesion of human monocytes to EC treated with the regenerated sample. EC were treated with samples for 18 h, followed by 6 h stimulation with TNF-α (0.5 ng/ml) and a static adhesion assay with fluorescence-labelled THP-1 human monocytes was performed. Adherent monocytes were measured in a plate fluorescence reader with 485 nm excitation and 530 nm emission wavelength. (C) The media from EC treated with the regenerated sample and activated with TNF-α was assayed to measure MCP-1 and IL-8 concentration by ELISA. Data were calculated as mean +/− SEM of 3 independent experiments. Data were reported as percentage of control (TNF-α activated EC); Statistical analysis was carried out using one-way ANOVA employing Dunnett correction for multiple comparisons. A statistical value of *P < 0.05 or greater was considered significant; $$$$ (p < 0.0001) vehicle vs control; ****P < 0.0001, ***P < 0.001 and **P < 0.01 treatments vs control (TNF-α activated EC). Additional file 2: Figure S2. Gene expression analysis of adhesion molecules in EC treated with the “Control Samples”. EC were treated with control sample (no *Enterococcus* in the fermentation process) (A), with *Enterococcus* strain PS063 (B) and with *Enterococcus* strain FHI0100 (C) for 18 h, followed by 6 h stimulation with TNF-α (0.5 ng/ml). RNA extraction and real-time PCR were performed as described in the Methods section. We used these two different *Enterococcus* strains as control samples because they could be compared to the strain used to produce the hydrolysate studied here (*Enterococcus* strain DPC763) since these have similar proteolytic profiles and base additions in the preparation process. As shown, these samples do not have any significant effect on adhesion molecules expression in EC, demonstrating that the effects of the hydrolysate studied here is due to the specific bioactive peptides generated by the bacterial fermentation with *Enterococcus* strain DPC763 and these effects are not due to a general hydrolysed material or pH effect. Additional file 3: Figure S3. Gene expression analysis of adhesion molecules in EC treated with fractions of sodium caseinate-derived hydrolysate. For fractions preparation the hydrolysate was resuspended in water and spun at 5000 rpm for 5 min, filtered through a 0.45um filter and then fractionated through a series of Millipore membranes of varying pore size (Merck Millipore, Billerica, MA) to generate specific protein fractions with enriched size ranges. EC were treated with fractions (150ug/ml) for 18 h, followed by 6 h stimulation with TNF-α (0.5 ng/ml). RNA extraction and real-time PCR were performed as described in the Methods section. Data were reported as percentage of control (TNF-α activated EC).Statistical analysis was carried out using one-way ANOVA employing Dunnett correction for multiple comparisons. A statistical value of *P < 0.05 or greater was considered significant; $$$$ (p < 0.0001) vehicle vs control; ***P < 0.001, **P < 0.01 and *P < 0.05 treatments vs control (TNF-α activated EC).
