# Supplementary figures and images for: Milk-derived bioactive peptides inhibit human endothelial-monocyte interactions via PPAR-γ dependent regulation of NF-κB
Source: J Inflamm (Lond). 2015 Jan 20;12:1. doi: 10.1186/s12950-014-0044-1 (PMC4308943; doi:10.1186/s12950-014-0044-1)

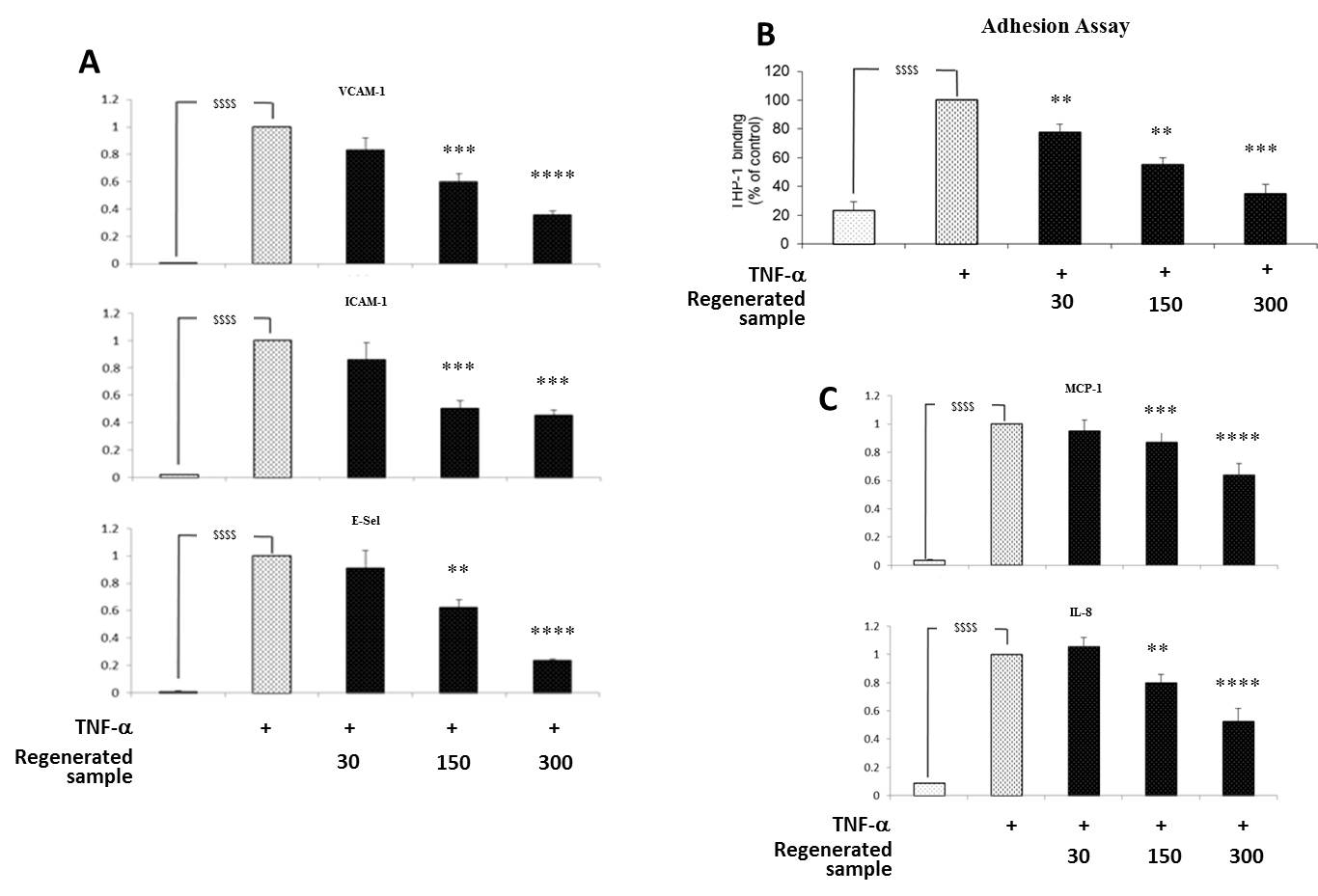

Supplement: Additional file 1: Figure S1. — Regenerated Sample of the Hydrolysate was obtained by incubating a different batch of sodium caseinate with a different batch of Enterococcus strain DPC763; the fermentation process was carried out as described in the Methods section. The incubation of EC with the regenerated sample showed similar results to the data obtained with the hydrolysate previously studied here, confirming the reproducibility of the procedure and of the study. (A) Gene expression analysis of adhesion molecules VCAM-1, ICAM-1 and E-Sel in EC treated with regenerated sample of the hydrolysate. EC were treated with samples for 18 h, followed by 6 h stimulation with TNF-α (0.5 ng/ml). (B) Adhesion of human monocytes to EC treated with the regenerated sample. EC were treated with samples for 18 h, followed by 6 h stimulation with TNF-α (0.5 ng/ml) and a static adhesion assay with fluorescence-labelled THP-1 human monocytes was performed. Adherent monocytes were measured in a plate fluorescence reader with 485 nm excitation and 530 nm emission wavelength. (C) The media from EC treated with the regenerated sample and activated with TNF-α was assayed to measure MCP-1 and IL-8 concentration by ELISA. Data were calculated as mean +/− SEM of 3 independent experiments. Data were reported as percentage of control (TNF-α activated EC); Statistical analysis was carried out using one-way ANOVA employing Dunnett correction for multiple comparisons. A statistical value of *P < 0.05 or greater was considered significant; $$$$ (p < 0.0001) vehicle vs control; ****P < 0.0001, ***P < 0.001 and **P < 0.01 treatments vs control (TNF-α activated EC). [file 12950_2014_44_MOESM1_ESM.jpeg]

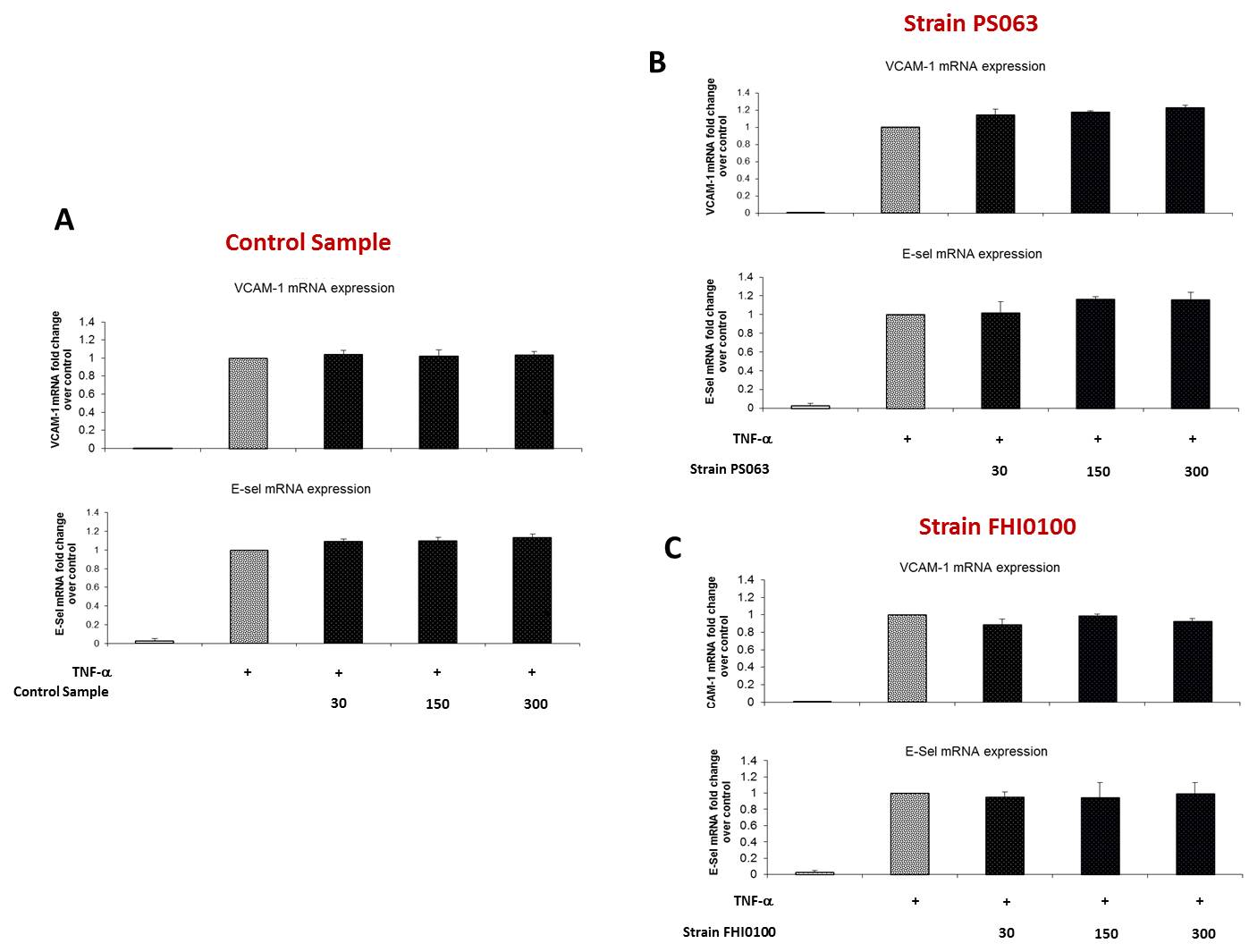

Supplement: Additional file 2: Figure S2. — Gene expression analysis of adhesion molecules in EC treated with the “Control Samples”. EC were treated with control sample (no Enterococcus in the fermentation process) (A), with Enterococcus strain PS063 (B) and with Enterococcus strain FHI0100 (C) for 18 h, followed by 6 h stimulation with TNF-α (0.5 ng/ml). RNA extraction and real-time PCR were performed as described in the Methods section. We used these two different Enterococcus strains as control samples because they could be compared to the strain used to produce the hydrolysate studied here (Enterococcus strain DPC763) since these have similar proteolytic profiles and base additions in the preparation process. As shown, these samples do not have any significant effect on adhesion molecules expression in EC, demonstrating that the effects of the hydrolysate studied here is due to the specific bioactive peptides generated by the bacterial fermentation with Enterococcus strain DPC763 and these effects are not due to a general hydrolysed material or pH effect. [file 12950_2014_44_MOESM2_ESM.jpeg]

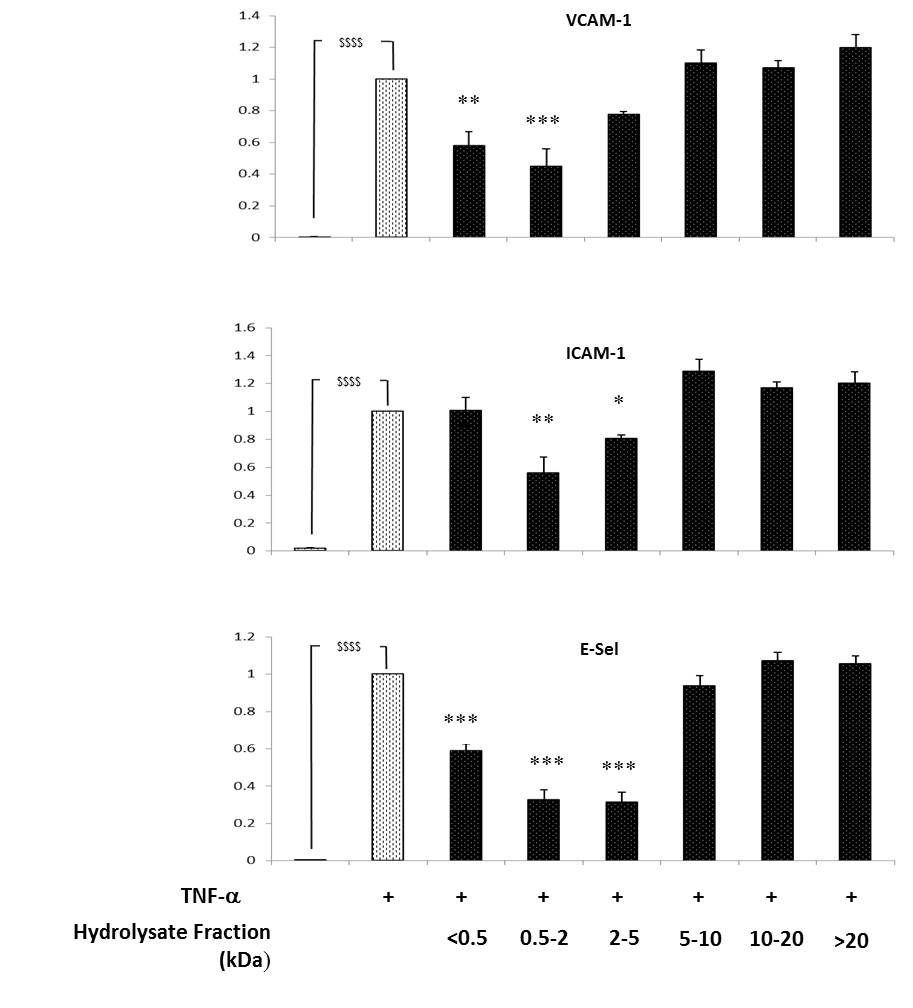

Supplement: Additional file 3: Figure S3. — Gene expression analysis of adhesion molecules in EC treated with fractions of sodium caseinate-derived hydrolysate. For fractions preparation the hydrolysate was resuspended in water and spun at 5000 rpm for 5 min, filtered through a 0.45um filter and then fractionated through a series of Millipore membranes of varying pore size (Merck Millipore, Billerica, MA) to generate specific protein fractions with enriched size ranges. EC were treated with fractions (150ug/ml) for 18 h, followed by 6 h stimulation with TNF-α (0.5 ng/ml). RNA extraction and real-time PCR were performed as described in the Methods section. Data were reported as percentage of control (TNF-α activated EC).Statistical analysis was carried out using one-way ANOVA employing Dunnett correction for multiple comparisons. A statistical value of *P < 0.05 or greater was considered significant; $$$$ (p < 0.0001) vehicle vs control; ***P < 0.001, **P < 0.01 and *P < 0.05 treatments vs control (TNF-α activated EC). [file 12950_2014_44_MOESM3_ESM.jpeg]
